# DKK3 and SERPINB5 as novel serum biomarkers for gastric cancer: facilitating the development of risk prediction models for gastric cancer

**DOI:** 10.3389/fonc.2025.1536491

**Published:** 2025-03-31

**Authors:** Yan-Yu Liu, Yan-Fang Fu, Wan-Yu Yang, Zheng Li, Qian Lu, Xin Su, Jin Shi, Si-Qi Wu, Di Liang, Yu-Tong He

**Affiliations:** ^1^ School of Public Health, Hebei Medical University, Shijiazhuang, China; ^2^ Cancer Institute, The Fourth Hospital of Hebei Medical University, Shijiazhuang, China

**Keywords:** gastric cancer, risk prediction, proteomics, biomarkers, screening

## Abstract

The existing gastric cancer (GC) risk prediction models based on biomarkers are limited. This study aims to identify new promising biomarkers for GC to develop a risk prediction model for effective assessment, screening, and early diagnosis. This study was conducted utilizing a large combined cohort for upper gastrointestinal cancer that was established in Hebei Province, China. General macro risk factors, Helicobacter pylori (H.pylori) infection status, and protein biomarkers were collected through questionnaire surveys and laboratory tests. Novel GC biomarkers were explored using data-independent acquisition (DIA) proteomics and enzyme-linked immunosorbent assay (ELISA). Multiple machine learning algorithms were used to identify key predictors for the GC risk prediction model, which was validated with an independent external cohort from multiple hospitals. A total of 530 participants aged 40 to 74 were analyzed, with 104 ultimately diagnosed with GC. Significant biomarkers in GC patients were identified by DIA combined ELISA, including elevated Keratin 7 (KRT7) and Mammary fibrostatin (SERPINB5) (P<0.001) and decreased Dickkopf-associated protein 3 (DKK3) (P<0.001). Factors such as sex, age, smoking status, alcohol consumption, family history of GC, H. pylori infection, DKK3 and SERPINB5 were used to create a multidimensional risk prediction model for GC. This model achieved an area under the curve (AUC) of 0.938 (95% confidence interval: 0.913-0.962). The risk prediction model developed in this study shows high accuracy and practical utility, serving as an effective preliminary screening tool for identifying high-risk individuals for GC.

## Introduction

Gastric cancer (GC) is the fifth most common cancer worldwide, with about 968,350 new cases annually, representing 4.9% of all new cancer diagnoses. China accounts for 37.1% of global GC incidence and 39.4% of related mortality (1). Despite advancements in diagnosis and treatment, patient prognosis remains unsatisfactory, with survival rates varying globally from 5% to 69% (2). In China, the five-year survival rate for GC patients is only 35.1% (3). Screening has been proven to be an effective approach to improve survival rates, and since the 21st century, China has implemented numerous screening programs for GC (4). However, these programs lack effective verification and evaluation. Meanwhile, GC screening programs rely mainly on endoscopy, but concerns regarding its invasive nature, the requirement for skilled endoscopists and pathologists, and the high costs in developing countries have been problems that have beset the prevention of GC (5). Therefore, accurate risk prediction is of significance as screening resources can be allocated more efficiently.

While some GC risk prediction models have been developed to support risk stratification strategies, there is a lack of comparative evaluation and no uniform conclusion, due to variations in study design, statistical methods, and model performance (6). Moreover, most diagnostic models suffer from inadequate sample sizes and rely solely on univariate analysis to select candidate variables, which may lead to the omission of important predictors. Additionally, comprehensive model evaluation is often neglected, and the vast majority of predictive models lack either internal or external validation, limiting their clinical applicability. Furthermore, most existing models are based solely on macro-epidemiological factors and known molecular markers. Apart from Helicobacter pylori (H. pylori), molecular markers of GC are largely unidentified, and the etiology underlying GC remains to be fully elucidated. Taken together, the published risk prediction models constructed based on GC molecular markers are extremely limited, particularly those based on prospective studies, and there is a critical need for well-performing biomarkers.

Proteomics has gradually been applied to the early diagnosis of GC due to its high serum proteomic content combined with systemic and local tumor features. The equipment for measuring serum protein is also very mature (7, 8). Exploring novel GC screening markers based on proteomics may improve efficiency in risk stratification and further optimize GC risk prediction models. Currently, some proteomic studies have been applied to the therapeutic targets and prognosis prediction of GC, but few have been applied to population risk stratification. Most of these studies lack control for possible confounding factors and multiple comparison correction or are limited by small sample size (9).

This study aims to identify promising new biomarkers for GC screening. And based on the new markers, we will develop and optimize a population-specific risk prediction model for GC in Hebei Province, and provide an effective assessment and screening method for the general population.

## Methods

### Study design

Two large screening cohorts for upper gastrointestinal cancer were established in Hebei Province, China. These two cohorts were combined to serve as the model derivation cohort for this study. The Cancer Hospital of the Chinese Academy of Medical Sciences initiated a project aimed at creating a large-scale population screening cohort for upper gastrointestinal cancer across six provinces: Henan, Hebei, Shandong, Jiangsu, Shanxi, and Hunan (10). This project successfully developed a multicenter screening cohort, database, and biobank encompassing 110,000 individuals. Participants eligible for this study were residents aged 40-74 years old, with no personal history of cancer and who had not undergone endoscopy in the preceding 3 years. As of 2019, the baseline survey was completed at all project sites within Hebei Province. Specifically, a total of 24,679 individuals participated in this study.

Additionally, another initiative has been conducting upper gastrointestinal cancer screenings as part of the Cancer Screening Program in Urban China (CanSPUC) in Hebei since 2012 (11). Individuals are screened using questionnaires and H. pylori tests, then were advised to undergo endoscopic examinations and provide five milliliters of venous blood for biomarker analysis. Participants were aged between 45 and 74 years and had no prior history of diagnosis or treatment for significant heart, brain, lung, or kidney dysfunctions, serious mental disorders, or cancer. We selected data from a total of 65,435 individuals within the CanSPUC cohort at baseline survey out of an initial pool of 210,381 individuals, which included participants from all eleven cities in Hebei Province.

**Figure 1:**
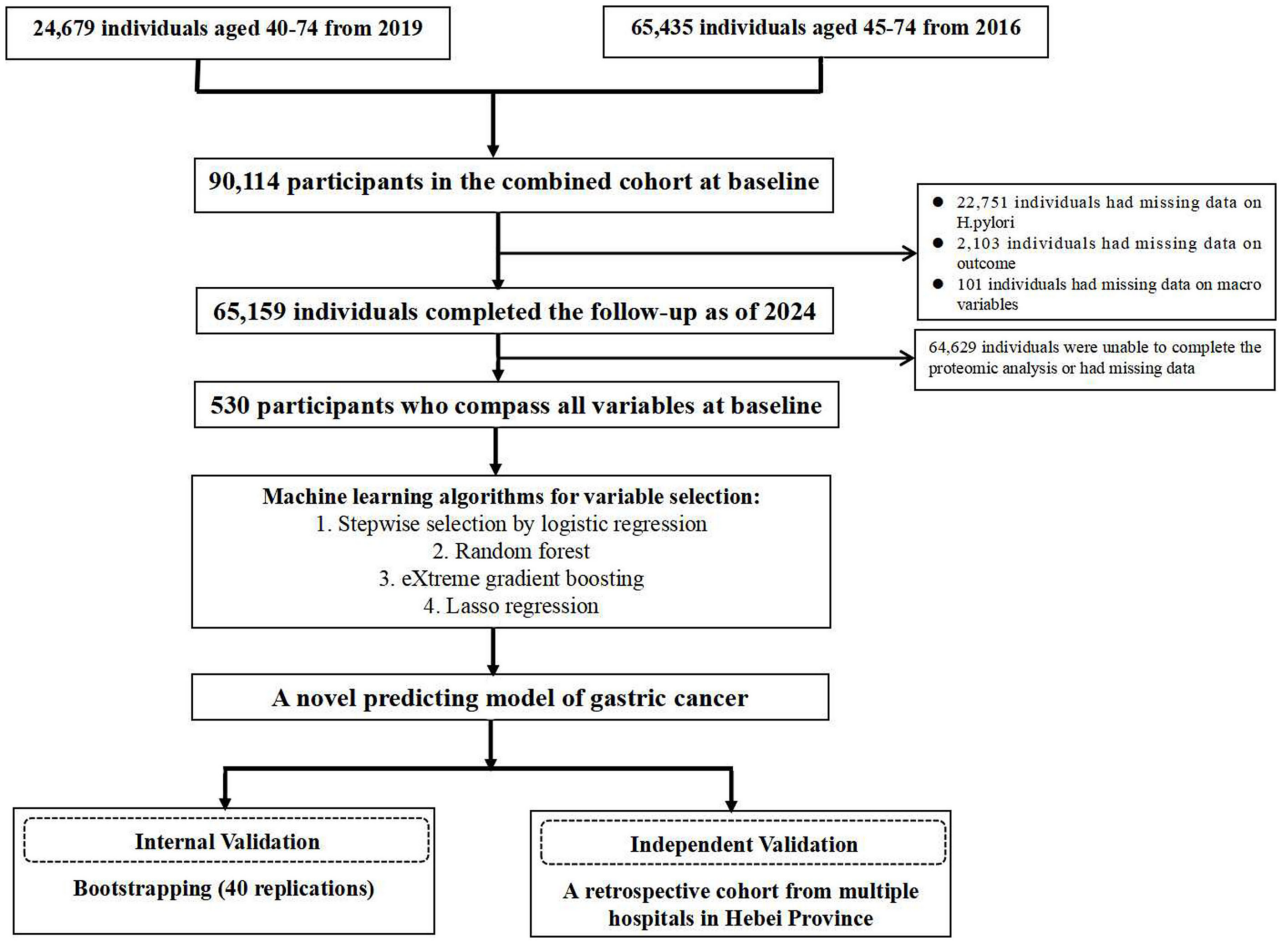
Flowchart of the entire research process.

For the purpose of independent verification, we utilized a retrospective cohort comprising clinical early-stage GC patients and healthy individuals from multiple hospitals in Hebei Province. This cohort included 81 gastric cancer patients and 210 healthy participants. The detailed study design and cohorts are depicted in **Figure 1**.

This study has been approved by the Ethics Committees of the Cancer Hospital, Chinese Academy of Medical Sciences and the Fourth Hospital of Hebei Medical University, in accordance with the principles of the Helsinki Declaration. All participants have signed informed consent forms.

### Definition of macro variables

The questionnaire primarily consisted of general demographic information such as age and sex, behavioral factors including smoking and drinking habits, frequency of consumption of a total of 10 diets such as fresh vegetables and fruits, history of digestive diseases, family history of tumors, and body mass index (BMI). All variables are included in the model as categorical variables. Age is divided into two groups based on whether they have reached the age of 60. BMI is divided into three groups based on the threshold values of 18.5 and 23.9 (kg/m^2^). Marital status was dichotomized into unmarried (separated, divorced, widowed, or never married) and married at the time of interview. Educational level was categorized as primary and below, secondary school, and college and above. Occupational exposure was categorized as absent and present. All dietary habits are categorized as either seldom or frequently based on self-reported questionnaire results from participants. All diseases history and family history are classified as present or absent. Smoking status was classified as smoker, ex-smoker, and non-smoker based on current smoking status and past smoking history. Drinking status was categorized as drinker and non-drinker based on the history of alcohol consumption.

### Preliminary experiment for differential protein screening

A preliminary case-control study was conducted to identify potential biomarkers by comparing serum protein profiles between early GC patients and healthy controls. Five cases of early clinical GC were collected, and healthy controls were matched 1:1 based on age and sex. The GC patients were recruited from the Fourth Hospital of Hebei Medical University, while the healthy controls were obtained from the hospital's health examination population. This pre-experiment aimed to identify differential proteins that could serve as candidate biomarkers for further validation in the main cohort study. The findings from this preliminary study will guide the subsequent validation within the main cohort.

The selection criteria for the early GC patients in the preliminary study were as follows: (1) age between 40 and 74 years; (2) histopathologically confirmed gastric cancer and clinically diagnosed as stage I; (3) no acute infections, allergies, or autoimmune diseases within the past three months that could affect blood biomarker expression; (4) no blood transfusions within the past three months; (5) normal liver and kidney function, as well as routine blood tests; (6) no history of other malignancies; and (7) no prior treatment with surgery, chemotherapy, or radiotherapy. The inclusion criteria for the healthy controls were as follows: (1) age between 40 and 74 years; (2) no history of malignancies or other major diseases; (3) no acute infections, allergies, or autoimmune diseases within the past three months that could affect blood biomarker expression; and (4) normal liver and kidney function, as well as routine blood tests.

Blood samples from GC patients who did not undergo chemoradiation were collected on an empty stomach before surgery and anesthesia. The serum was separated by centrifugation at 2000 rpm for 10 minutes after the coagulating tube was placed for 30 minutes as pre-experimental samples.

### Data-independent acquisition proteomic analysis

Data-independent acquisition (DIA) proteomic analysis was conducted on serum samples from both cases and controls in order to identify differential proteins. The detailed DIA process followed the procedures outlined below, including protein extraction, determination of protein concentration, protease hydrolysis, desalination, peptide classification, establishment of a DDA reference map, data collection using DIA methodology, high-precision mass spectrometry detection, and qualitative database search for bioinformatics analysis (12). The DIA analysis was performed using a Q Exactive HF-X Hybrid Quadrupole-Orbitrap Mass Spectrometer (Thermo Fisher Scientific) coupled with a high-performance liquid chromatography system (EASY nLC 1200, Thermo Fisher Scientific). Quality control measures were implemented for each experimental session. These measures included instrument calibration, the use of internal standards, data replication, and peptide identification validation against a decoy database to control the false discovery rate (FDR) below 1%. Identification of target proteins involved a combination of domestic and international research findings as well as enrichment analysis of GO (Gene Ontology) and KEGG (Kyoto Encyclopedia of Genes and Genomes) databases. Given the complexity of biological samples and the potential for non-normal distributions in protein expression levels, differential proteins were analyzed using a two-sided Wilcoxon rank-sum test (*p* < 0.05 and fold change > 2 or <0.5). Significantly enriched GO functions and KEGG pathways were determined using Fisher’s exact test followed by Benjamini–Hochberg correction with *p* < 0.05.

### Enzyme-linked immunosorbent assay

The diagnostic validity of the new biomarkers for GC was confirmed through enzyme-linked immunosorbent assay (ELISA) in the serum samples of derivation cohort. The levels of the new biomarkers in both case and control groups were determined using the corresponding proteins ELISA kit (Jianglai, Shanghai), following the manufacturer’s instructions. Briefly, 100 µl of serum diluent was added to a 96-well plate coated with human target protein. Then 100 µl of HRP-conjugated mixed solution was added to each well, and the plate was incubated for 0.5 h at 37°C. After several washes, the color reaction was developed with the substrate solution and blocked with stop solution. The optical densities were measured at 450 nm.

### Definition of outcome

The study outcome was a confirmed diagnosis of GC, as identified by the International Statistical Classification of Disease-10 code C16. The identification was achieved through screening detection, active follow-up, and passive follow-up. Participants with positive screening results were subsequently followed up by phone or retrieval of medical records, and the entire cohort population was also passively matched using the population-based Hebei Cancer Registry and Cause of Death Database until June 30, 2024 to obtain final diagnoses and outcomes.

### Development of cancer risk prediction models

The predictive model was developed in the derivation cohort and then validated in the independent validation cohort. Using GC occurrence as the dependent variable and the selected variables from the derivation cohort as independent variables, the GC risk prediction models were developed using multiple logistic regression. All potential macro risk factors are initially included in the underlying models. Four machine learning (ML) algorithms - logistic regression (LR), random forest (RF), lasso regression and eXtreme gradient boosting (XGBoost) - were employed to screen the variables ultimately included in the model. LR utilized a stepwise regression method for variable screening. RF used the reduction of average accuracy rate as the evaluation index and ranked the importance of variables. Lasso regression employed minimum mean to filter variables, while XGBoost ranked variables by importance. Predictive variables meeting the criterion of *p* < 0.05 were selected for inclusion in the model. Variable selection was achieved using R packages "MASS", "randomForest", "lasso" and "XGBoost".

Based on proteomic analysis, we investigated and identified novel GC markers. Subsequently, we integrated these markers with all variables in the cohort to identify predictors for GC through machine learning techniques. Therefore, the GC risk prediction model constructed by the final model contains the following risk factors:


$$Logit\;P\;=\;\beta_{0}+\;\beta_{1}*\;macro\;risk\;factors\;+\;\beta_{2}*\;H.pylori\;+\;\beta_{3}*\;novel\;markers$$

Where *Logit P* is the expected the risk of incident GC; *β_0_* is the intercept. *β_1_*, *β_2_*, and *β_3_* are the coefficients of each variable.

### Statistical analysis

The descriptive statistics of baseline characteristics are presented according to the risk of GC. Categorical variables are displayed as frequencies and percentages. Chi-square tests were used for comparing differences in categorical variables. All these available factors were comprehensively screened in our prediction model to enhance its accuracy and to identify potential unrecognized risk factors for GC. The overall performance of the models was assessed based on the area under curve (AUC), while their calibration was evaluated using Hosmer-Lemeshow chi-square test results (H-L χ^2^). The prediction models were internally validated using bootstrapping (40 replications).

Two-sided tests were used in all studies, and *p* < 0.05 was considered to be statistically significant. Statistical analyses were performed using R (v.4.2.0) software.

## Results

### Characteristics of the study population

The combined cohort consists of 90,114 individuals. After excluding 24,955 participants with missing key variables or those who were lost to follow-up regarding outcomes, we conducted ELISA assays on preserved serum samples from 530 individuals of the remaining population. During the study period, a total of 530 eligible participants were enrolled in the derivation cohort, among whom 104 eventually developed GC. The characteristics of all participants are presented in **Table 1**. The majority of the GC participants were aged 60 and above, accounting for 67.3%. Additionally, males comprised 72.1% of the GC participant group, and 52.9% of the GC participants were overweight or obese. A total of 291 participants, comprising 81 patients with GC and 210 healthy individuals, were included for independent validation. The baseline information for the independent validation cohort can be found in **Supplementary Table 1**.

**Table 1:** Baseline characteristics of the derivation population.

Characteristics	Levels	Non-gastric cancer (N=426)	Gastric cancer (N=104)	p
Sex (%)	Male	204 (47.9)	75 (72.1)	<0.001
	Female	222 (52.1)	29 (27.9)	
Age (years) (%)	<60	228 (53.5)	34 (32.7)	<0.001
	≥60	198 (46.5)	70 (67.3)	
BMI (%)	<18.5	2 (0.5)	3 (2.9)	0.066
	18.5-23.9	182 (42.7)	46 (44.2)	
	≥23.9	242 (56.8)	55 (52.9)	
Marriage (%)	Unmarried	16 (3.8)	4 (3.8)	1.000
	Married	410 (96.2)	100 (96.2)	
Education (%)	Primary and below	177 (41.5)	66 (63.5)	<0.001
	Secondary school	145 (34.0)	38 (36.5)	
	College and above	104 (24.4)	0 (0.0)	
Occupational exposure (%)	Absent	334 (78.4)	93 (89.4)	0.016
	Present	92 (21.6)	11 (10.6)	
Smoking status (%)	Never smoker	288 (67.6)	57 (54.8)	0.002
	Smoker	99 (23.2)	28 (26.9)	
	Ex-smoker	39 (9.2)	19 (18.3)	
Drinking status (%)	Absent	289 (67.8)	53 (51.0)	0.001
	Present	137 (32.2)	51 (49.0)	
Vegetables (%)	Seldom	98 (23.0)	3 (2.9)	<0.001
	Frequently	328 (77.0)	101 (97.1)	
Fruits (%)	Seldom	169 (39.7)	17 (16.3)	<0.001
	Frequently	257 (60.3)	87 (83.7)	
Milk, meat and egg production (%)	Seldom	202 (47.4)	19 (18.3)	<0.001
	Frequently	224 (52.6)	85 (81.7)	
Pickled food (%)	Seldom	252 (59.2)	92 (88.5)	<0.001
	Frequently	174 (40.8)	12 (11.5)	
Fried food (%)	Seldom	313 (73.5)	99 (95.2)	<0.001
	Frequently	113 (26.5)	5 (4.8)	
Hot diet (%)	Seldom	210 (49.3)	92 (88.5)	<0.001
	Frequently	216 (50.7)	12 (11.5)	
History of UDTDs (%)	Absent	219 (51.4)	87 (83.7)	<0.001
	Present	207 (48.6)	17 (16.3)	
Diabetes (%)	Absent	381 (89.4)	100 (96.2)	0.053
	Present	45 (10.6)	4 (3.8)	
Hypertension (%)	Absent	280 (65.7)	77 (74.0)	0.133
	Present	146 (34.3)	27 (26.0)	
Hyperlipemia (%)	Absent	292 (68.5)	100 (96.2)	<0.001
	Present	134 (31.5)	4 (3.8)	
Family history of cancer (%)	Absent	193 (45.3)	78 (75.0)	<0.001
	Present	233 (54.7)	26 (25.0)	
Family history of gastric cancer (%)	Absent	315 (73.9)	67 (64.4)	0.617
	Present	111 (26.1)	37 (35.6)	
H.Pylori (%)	Negative	297 (69.7)	54 (51.9)	0.001
	Positive	129 (30.3)	50 (48.1)	
KRT7 (%)	Negative	252 (59.2)	41 (39.4)	<0.001
	Positive	174 (40.8)	63 (60.6)	
DKK3 (%)	Negative	148 (34.7)	68 (65.4)	<0.001
	Positive	278 (65.3)	36 (34.6)	
SERPINB5 (%)	Negative	328 (77.0)	21 (20.2)	<0.001
	Positive	98 (23.0)	83 (79.8)	

BMI, body mass index; History of UDTSs, History of upper digestive tract diseases; H.Pylori, Helicobacter pylori; KET 7, Keratin 7; DKK 3, Dickkopf-associated protein 3; SERPINB5, Mammary fibrostatin.

### Selection of novel markers for GC based on proteomics

**Figure 2:**
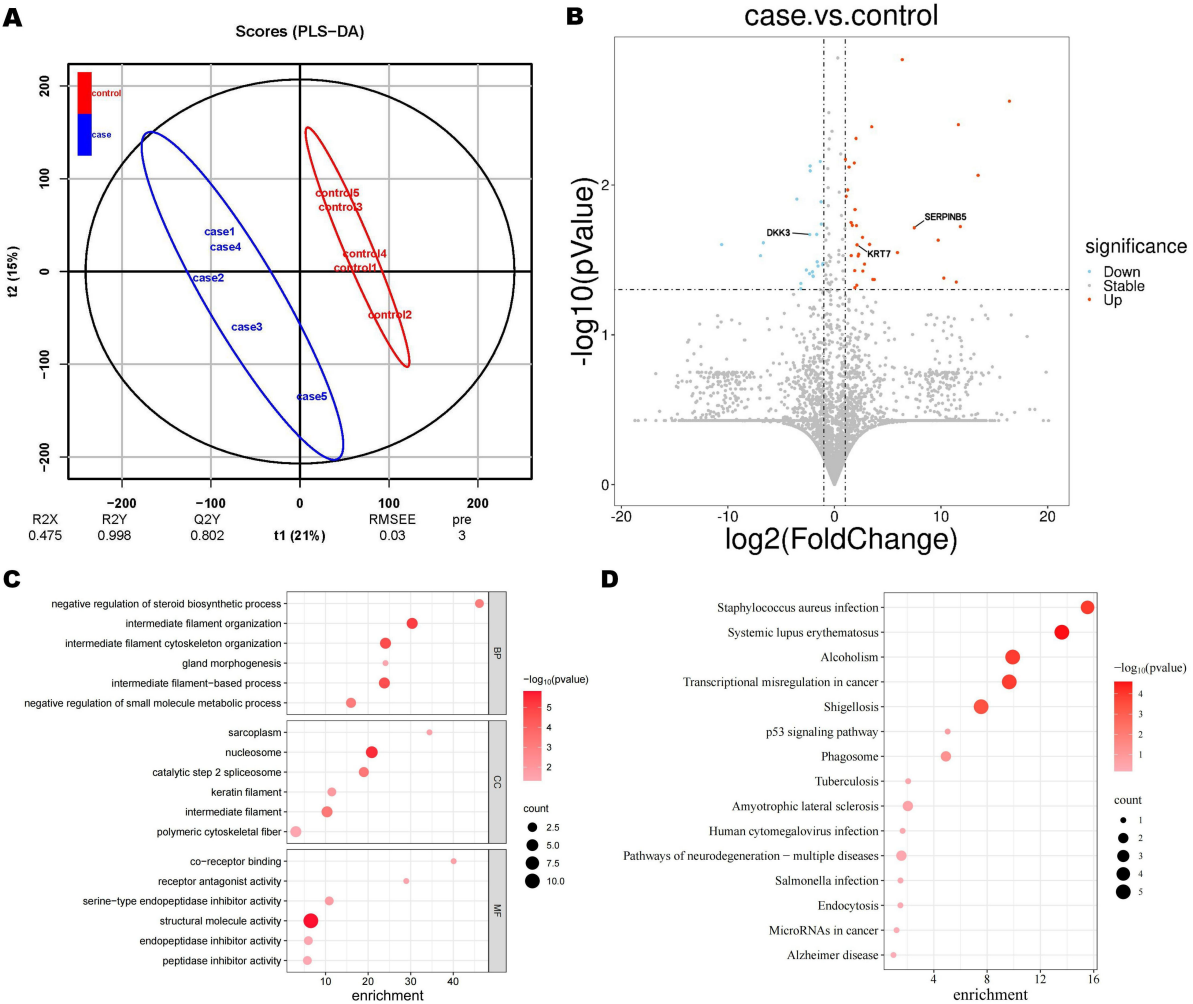
Proteomic findings. **(A)** Partial least square discriminant analysis (PLS-DA) score. **(B)** Volcano plot of differential proteins. The blue circles on the left indicate down-regulated proteins, while the red circles on the right represent up-regulated proteins. The x-axis represents fold change, and the y-axis represents P value. The two-sided Wilcoxon rank-sum test was employed to identify proteins with an expression fold change of > 2 (either upregulated or down-regulated) and a p-value < 0.05 as differentially expressed proteins. Significantly enriched GO terms **(C)** and KEGG terms **(D)**. Dot plots and enrichment plots display biological processes along the vertical axis, with circle size indicating gene counts. The depth of colors represents the P value.

Partial least squares discriminant analysis (PLS-DA) score plots revealed a significant disparity in proteomic components between GC patients and non-GC individuals (**Figure 2A**). A total of 4837 proteins were identified using DIA. Among them, 60 proteins showed differential expression between the case and control groups, with 37 up-regulated and 23 down-regulated proteins. A volcano plot was created to visually display the differential protein expression patterns (**Figure 2B**).

In the GO enrichment analysis of case and control, differential proteins were significantly enriched in biological functions such as intermediate filament organization, nucleolus, and chromatin structural components (**Figure 2C**). While in the KEGG enrichment analysis, pathways related to transcriptional dysregulation in cancer were significantly enriched (**Figure 2D**). Based on previous research and taking into account the enrichment findings of GO and KEGG, we have identified 3 proteins from the pool of differential proteins for further ELISA analysis (13–15). These proteins are Keratin 7 (KRT 7), Dickkopf-associated protein 3 (DKK 3), and Mammary fibrostatin (Maspin / SERPINB5).

### Validation of new markers by ELISA

**Figure 3:**
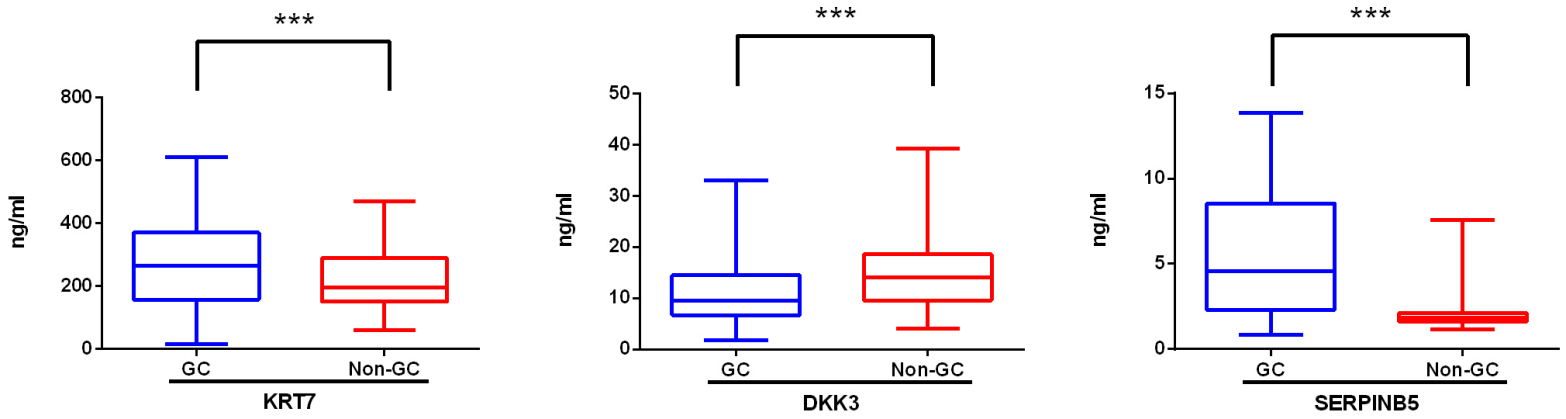
Levels of novel biomarkers in the serum of gastric cancer patients (GC) and non-gastric cancer individuals (non-GC). The expression level of KRT 7, DKK3, and SERPINB5 in serum samples from GC (n=104) and non-GC (n=426) individuals was measured with ELISA. When the P value is less than 0.001, three asterisks (***) are used to indicate statistical significance. KRT 7, Keratin 7; DKK 3, Dickkopf-associated protein 3; SERPINB5, Mammary fibrostatin.

We further assessed the expression levels of DKK3, KRT7, and SERPINB5 in serum samples from both GC patients and non-GC individuals using ELISA. In comparison to the non-GC samples, we observed a three-fold increase in the SERPINB5 level in GC samples (mean: 8.56 ng/ml vs 2.06 ng/ml), a significant increase in the KRT7 level (mean: 275.88 ng/ml vs 217.14 ng/ml), and a decrease in the DKK3 level (mean: 11.00 ng/ml vs 15.47 ng/ml) (**Figure 3**). Based on the median concentrations of biomarkers measured by ELISA, the three biomarkers are categorized into negative and positive groups. The categorical variables of the biomarkers are incorporated into the prediction models for variable screening.

### Variable selection and performance of the model

**Figure 4:**
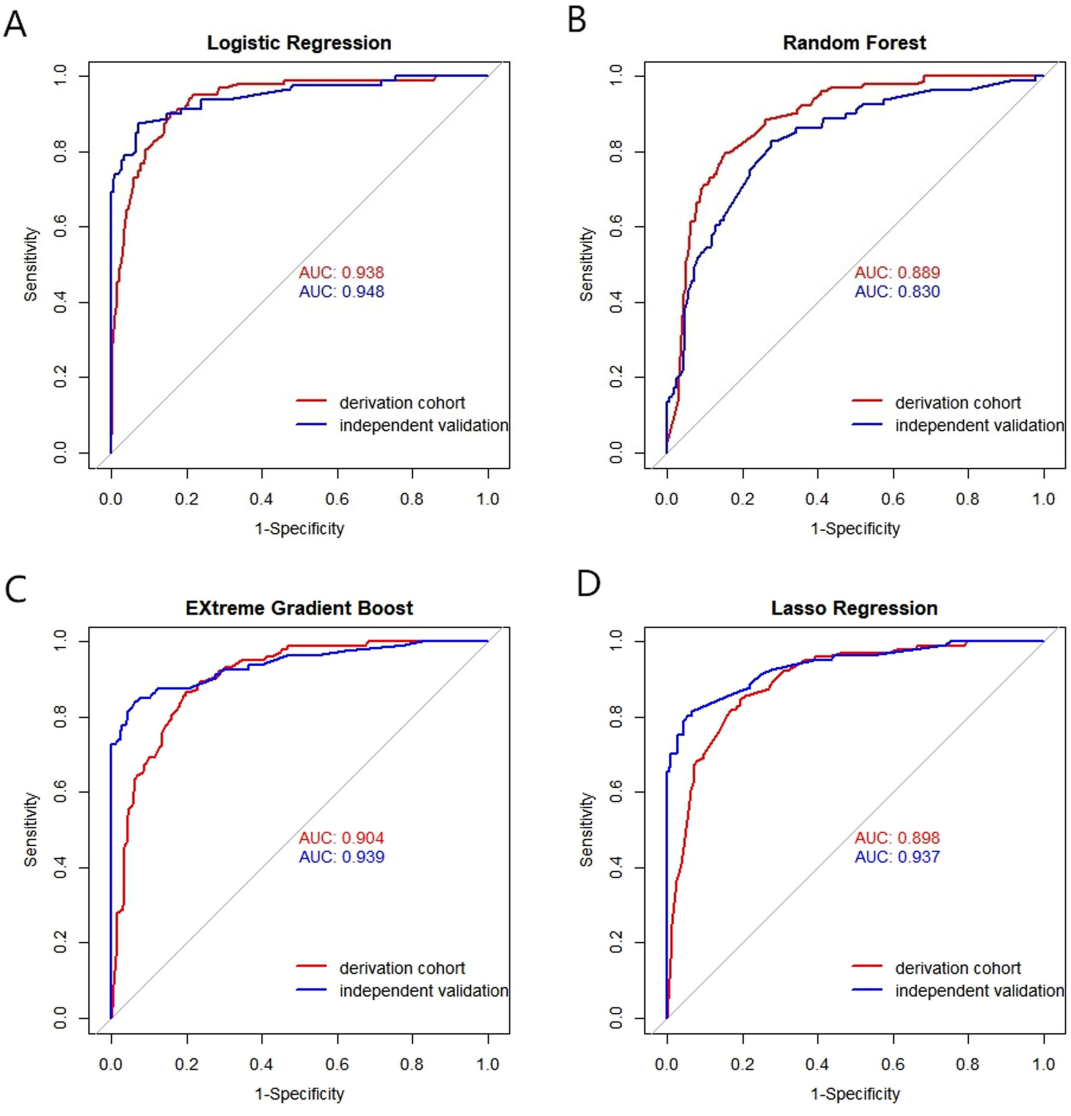
Receiver operating characteristic (ROC) curves of the models by four machine algorithms in derivation and validation cohort. **(A)** Logistic Regression, **(B)** Random Forest, **(C)** EXtreme Gradient Boost, **(D)** Lasso Regression. X-axis: False positive rate (1-specificity); Y-axis: True positive rate (sensitivity). AUC: Area under the curve, indicating model discrimination ability, with higher values suggesting better performance in distinguishing gastric cancer patients.

The discrimination performance metrics of the prediction models in derivation and independent validation cohorts are presented in **Table 2**. In the derivation cohort, the stepwise selection by LR model attained the highest AUC of 0.938 (95% confidence interval (CI): 0.913-0.962). All the ML algorithms showed statistically significant results. In independent validation, the LR model achieved a high AUC of 0.948 (95% CI: 0.916-0.980). Its AUC was significantly higher than the RF model, but comparable to other ML models with AUC in the range of 0.937-0.939. ROC curves of all prediction models are shown in **Figure 4**.

**Table 2:** Discrimination performance of gastric cancer risk prediction models derived from four machine learning algorithms.

Algorithm used in model development	Logistic regression	Random forest	eXtreme gradient boosting	Lasso regression
Factors	Sex, Age group, Smoke, Drink, FGH, H.Pylori, DKK3, SERPINB5	BMI, Education, Smoke, MME production, History of UDTSs, DKK3, SERPINB5	Sex, Age group, Smoke, BMI, Education, H.Pylori, SERPINB5	Sex, Age group, Smoke, Vegetables, Pickled food, H.Pylori, DKK3
Derivation cohort				
AUC (95% CI)	0.938 (0.913-0.962)	0.889 (0.857-0.921)	0.904 (0.876-0.933)	0.898 (0.867-0.930)
Sensitivity	0.843	0.847	0.801	0.805
Specificity	0.904	0.798	0.865	0.846
PPV	0.973	0.945	0.961	0.955
NPV	0.584	0.561	0.514	0.515
Kappa	0.619	0.557	0.529	0.534
Accuracy	0.855	0.838	0.813	0.813
Independent validation				
AUC (95% CI)	0.948 (0.916-0.980)	0.830 (0.776-0.883)	0.939 (0.904-0.974)	0.937 (0.903-0.971)
Sensitivity	0.929	0.724	0.945	0.948
Specificity	0.877	0.827	0.827	0.803
PPV	0.951	0.916	0.934	0.936
NPV	0.826	0.536	0.848	0.855
Kappa	0.790	0.472	0.776	0.765
Accuracy	0.914	0.753	0.911	0.907

FGH, Family history of gastric cancer; BMI, body mass index; MME production, Milk, meat and egg production; History of UDTSs, History of upper digestive tract diseases; H.Pylori, Helicobacter pylori; DKK 3, Dickkopf-associated protein 3; SERPINB5, Mammary fibrostatin; AUC, area under the receiver operating characteristic curve; CI, confidence interval; NPV, negative predictive value; PPV, positive predictive value.

Given that the LR model demonstrated superior performance compared to other ML models in both derivation and independent validation population, the final variables included in the prediction model were selected using logistic stepwise regression. These variables encompass sex, age group, smoking status, drinking status, family history of GC, H.pylori, DKK3, and SERPINB5. The association of these predictors with GC are detailed in **Table 3**. The findings suggest that the positivity of SERPINB5 are independent risk factors for GC, whereas DKK3 positivity is an independent protective factor against GC.

**Table 3:** Association between the selected predictors and the risk of gastric cancer.

Characteristics	Univariate		Multivariate	
	OR (95%CI)	P value	OR (95%CI)	P value
Sex		< 0.001		< 0.001
Male	1.00		1.00	
Female	0.36 (0.22-0.57)		0.28 (0.14-0.54)	
Age(years)		< 0.001		0.007
< 60	1.00		1.00	
≥ 60	2.37 (1.51-3.73)		4.08 (2.08-8.02)	
Smoking status		0.013		0.164
Never smoker	1.00		1.00	
Smoker	1.43 (0.86-2.37)		1.40 (0.78-2.52)	
Ex-smoker	2.46 (1.33-4.57)		2.10 (0.94-4.67)	
Drinking status		0.001		0.078
Never-drinker	1.00		1.00	
Drinker	2.03 (1.31-3.14)		1.62 (0.95-2.78)	
Family history of gastric cancer		0.053		0.049
Absent	1.00		1.00	
Present	1.57 (0.99-2.47)		1.23 (1.00-5.46)	
H. pylori		0.001		0.037
Negative	1.00		1.00	
Positive	2.13 (1.38-3.30)		2.04 (1.05-4.00)	
DKK 3		< 0.001		< 0.001
Negative	1.00		1.00	
Positive	0.28 (0.18-0.44)		0.24 (0.13-0.43)	
SERPINB5		< 0.001		< 0.001
Negative	1.00		1.00	
Positive	13.23(7.80-22.46)		14.57 (7.80-27.19)	

BMI, body mass index; H.Pylori, Helicobacter pylori; DKK 3, Dickkopf-associated protein 3; SERPINB5, Mammary fibrostatin; OR, odds ratio; CI, confidence interval.

**Figure 5:**
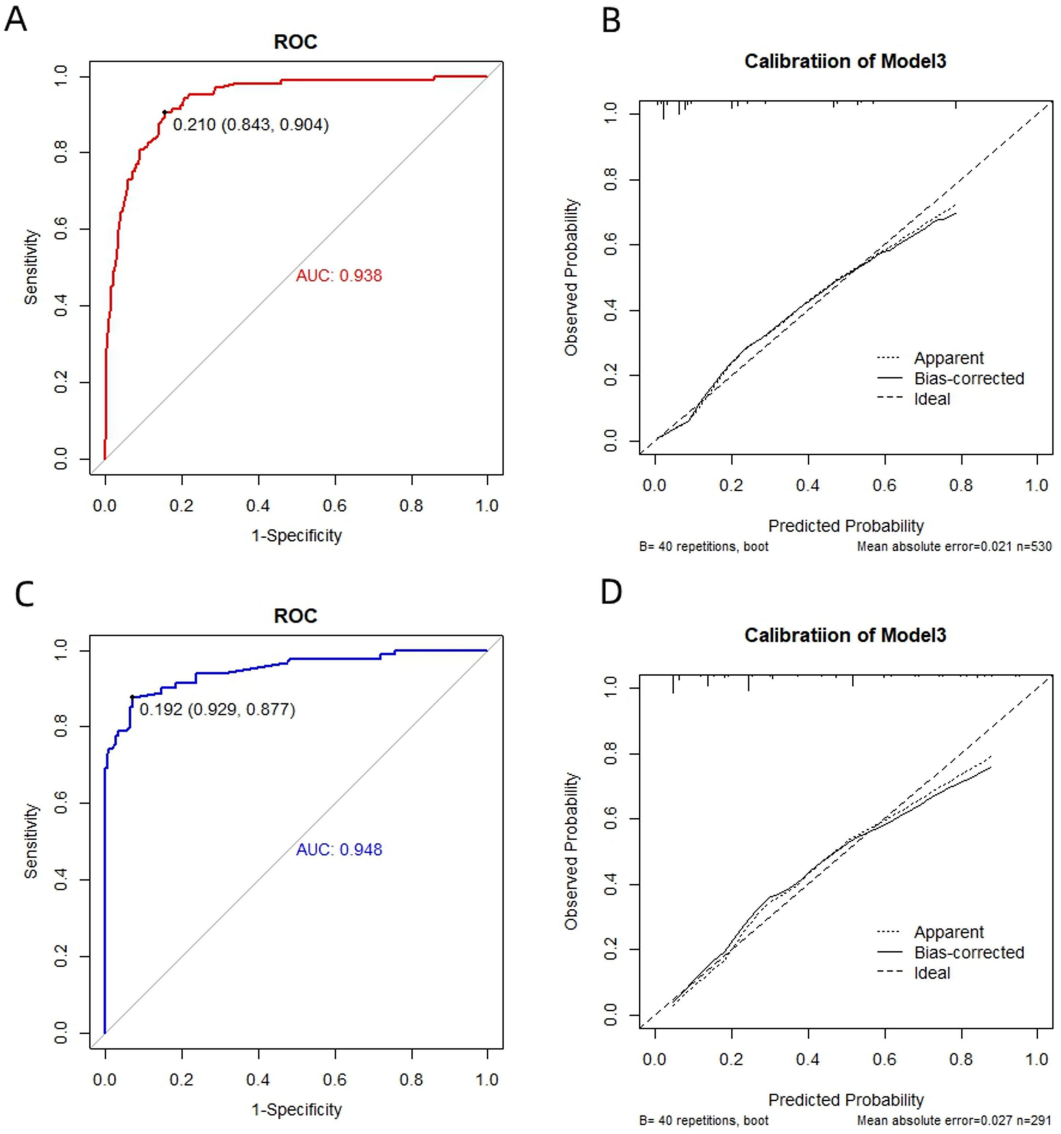
Receiver operating characteristic (ROC) and calibration curves for prediction model in derivation **(A, B)** and validation **(C, D)** cohort.

The ROC and calibration curves for the final prediction model in both derivation and validation cohort are shown in **Figure 5**. The predictive model demonstrates excellent performance, with an AUC ranging from 0.938 to 0.948. Additionally, the sensitivity and specificity have achieved values of 0.843-0.929 and 0.904-0.877, respectively.

## Discussion

To the best of our knowledge, this study represents the first attempt to develop a multidimensional GC risk prediction model that includes macro GC factors, identified GC markers, and novel GC biomarkers simultaneously. The multidimensional model in our study demonstrated strong performance in both derivation and validation cohorts, demonstrating its ability to identify high-risk patients for GC. As a result, this risk prediction model can serve as an initial pre-screening tool for gastroscopy to identify individuals at elevated risk of GC in Hebei Province.

Currently, numerous GC risk prediction models have been developed (6). However, due to variations in study design, statistical methods, and model performance, there is no standardized prediction rule for GC. Additionally, most diagnostic models lack an adequate sample size, select candidate variables through univariate analysis, or fail to undergo a comprehensive model evaluation. Furthermore, the vast majority of predictive models lack internal or external validation (16, 17). Our model is constructed based on a large combined cohort of individuals, adhering to a temporal sequence of cause and effect, with thorough internal and external validation. Various ML algorithms were employed to sift through candidate predictors from all questionnaire data, and the final chosen predictor circumvented the information loss typically associated with the traditional single-factor to multi-factor screening process.

In this study, the developed model by various ML algorithms demonstrated good performance metrics that included known GC key risk factors in the analysis. These results were both internally and externally validated, indicating that the prediction rule is robust and effective. The macro variables included in the model of this study are sex, age, smoking status, drinking status, and family history of GC. All of these factors have been confirmed to be closely related to the occurrence and development of GC (18). A positive family history of GC in first-degree relatives is a known risk factor for GC (19). Previous studies have also indicated that a patient with a positive family history is at increased risk for developing gastric prelesions (20). Similarly, smoking and drinking have been identified as risk factors for GC according to several meta-analyses (21, 22).

In most studies that only consider general demographic factors in building models, there are also some models that take into account laboratory measures, but most of them only focus on routine tests for H. pylori infection and pepsinogen (23, 24). Protein and gene tests are commonly utilized in basic research of GC, rather than in the development of risk prediction models. Currently, there is a scarcity of studies that have incorporated proteomics into GC risk prediction models. In traditional research, basic research and clinical application often operate separately, with biomarker discovery and model development as distinct stages (25, 26). Our approach differs by integrating biomarker discovery into the model construction process, allowing for concurrent validation of biomarker utility. On one hand, most studies only conduct prognostic analysis, making it challenging to predict disease risk due to difficulties in cohort construction and lack of a control group (27–29). On the other hand, marker discovery in some studies is tissue-based, posing challenges in sample acquisition and complex detection techniques which hinder their application to population screening and risk stratification (30).

In our study, we also took into consideration previously known H. pylori infections. It is widely recognized that H. pylori infection is the most significant risk factor for GC (31, 32). Since the discovery of H. pylori, numerous studies have established a link between H. pylori and GC as well as its precursors (33–35). Our study found that Helicobacter pylori infection was independently associated with the development of GC.

At the same time, the risk prediction model involving proteomics primarily focuses on typical carcinoembryonic antigens (36). It is crucial to identify additional biomarkers in the development of GC. A study investigated the humoral response of nearly all H. pylori immune proteome (1,527 proteins) in 50 GC cases and 50 control patients, and subsequently developed a GC prediction model. The findings revealed that the model, which incorporates four antibody proteins, achieved an AUC of 0.73 in distinguishing GC from control (37). Another study conducted in South Korea investigated blood-derived protein biomarkers for various types of cancer. Prediction models for six different types of cancer were developed using a panel of 12 blood proteins, with carcinoembryonic antigen being the main component. The AUC for the prediction model of GC was found to be 0.97 (38). However, it is important to note that this model only utilizes blood markers and does not take into account general demographic factors. While models that use only biomarkers may achieve greater accuracy in a controlled setting, integrated models that combine biomarkers with population variables, such as our study, can provide better generality for population-level implementation.

Our research has identified there novel biomarkers that exhibit significant differences between GC patients and non-GC individuals. However, following machine learning variable selection, only DKK3 and SERPINB5 were incorporated into the GC risk prediction model. This inclusion markedly enhanced the predictive performance of the model, achieving an AUC of 0.938. KRT 7, also known as cytokeratin-7 (CK-7), is the primary component of the intermediate filament cytoskeleton. Studies indicate that KRT7 is associated with cancer cell behaviors such as proliferation, migration, and invasion (39, 40). Particularly, KRT7 has also been confirmed to be significantly up-regulated in GC tissues and cell lines (13). Knockdown of KRT7 impairs GC cell proliferation and migration, and its activation in GC cells is driven by FOXA1 transcription, which enhances these processes (41, 42). However, after adjusting for other protein markers, H.pylori, and macro variables, KRT7 was not included in the final model. In the future, there may be more additional biomarkers to develop novel GC models, and KRT7 warrants further investigation.

Another two biomarkers are also supported by other foundational studies and have a certain biological rationale. Mammary fibrostatin (Maspin), or SERPINB5, is a member of the serine protease inhibitor superfamily. It is involved in regulating protein disassembly and has been implicated in various cancers, including colorectal and gallbladder cancers (43, 44). SERPINB5 has been confirmed as a novel serum diagnostic biomarker for high-grade intraepithelial neoplasia in GC and is involved in macrophage phenotype regulation (14). DKK 3, encoded by the DKK3 gene, plays critical roles in development, stem cell differentiation, and tissue homeostasis, and has immunomodulatory functions (45). DKK3 is a potential tumor suppressor, with downregulation observed in cancers such as prostate and ovarian cancer (46, 47). Previous studies have shown that reducing DKK3 enhances the migration and invasion of GC cells, which are consistent with our own findings. DKK3 regulates multiple pathways to suppress GC occurrence and progression (15, 48, 49). These biomarkers provide promising approaches for improving the performance of GC risk prediction model and understanding disease mechanisms.

Recent studies have elucidated the potential mechanisms of various molecular markers, including m1A-modified genes, miRNA-CD molecule interactions, and peptides encoded by lncRNAs, in the development, progression, and metastasis of gastrointestinal cancers (50–53). These markers are closely associated with key signaling pathways and influence the invasiveness and metastatic potential of tumors by modulating immune responses and cellular metabolism, showing high potential for predicting the occurrence and progression of gastric cancer. Future research should further explore their specific roles in the tumor microenvironment and integrate clinical samples with multi-omics data to identify additional potential markers and therapeutic targets, thereby advancing the precision diagnosis and treatment of gastrointestinal cancers.

The prediction rule developed in the novel GC model has good discrimination with an AUC of 0.938 in the derivation cohort and high sensitivity (84.3%). However, there are several potential limitations in the present study. Firstly, the serum samples were collected prior to the onset of the disease, and some GC patients were concurrently experiencing other gastric conditions at that time. This may have influenced the levels of serum biomarkers, thereby impacting the applicability of the GC prediction model we developed. However, previous research addressing this issue has indicated that stomach cancer cases accompanied by other gastric disorders are more likely to adhere to predictive guidelines and undergo endoscopy compared to those with isolated GC (54). Additionally, our questionnaire relied on self-reported data, and variables such as dietary habits could not be quantified accurately, which may introduce certain biases into our findings. However, our data collection was conducted by trained investigators following a standardized protocol to minimize potential biases. And the reliability of our data was internally verified through bootstrapping and confirmed in an independent external validation cohort, demonstrating the stability of our model. Moreover, the questionnaires used in this study are well-established and have been validated in multiple prior studies (55). Finally, our model is limited by the number of blood specimens and only included participants from Hebei province. However, the model developed in this study remains applicable for predicting the risk of GC in China and other Asian countries to a certain extent. This applicability is supported by the fact that over 90% of China's population is Han ethnic, which shares similar dietary habits and lifestyles with residents from other Asian nations such as Japan, Korea, and Singapore. Additionally, it provides foundational data for future cross-regional comparative studies. Future research should focus on verification and implementation in larger and diverse populations.

In conclusion, the risk prediction model established and validated in this study has shown good identification effectiveness for the high-risk population of GC in Hebei Province. Therefore, it can serve as an accurate and cost-effective initial large-scale pre-screening tool to improve the detection rate of GC, reduce unnecessary invasive screening and diagnosis, and thus enhance secondary prevention of GC. In the future, this screening strategy can be extended to validate and test its feasibility in a larger population nationwide.

## Data Availability

The raw data supporting the conclusions of this article will be made available by the authors, without undue reservation.
