# Supplementary material for: DKK3 and SERPINB5 as novel serum biomarkers for gastric cancer: facilitating the development of risk prediction models for gastric cancer
Source: Front Oncol. 2025 Mar 31;15:1536491. doi: 10.3389/fonc.2025.1536491 (PMC11994446; doi:10.3389/fonc.2025.1536491)
Supplement: Supplementary file 1 [file Table1.docx]

Supplementary table 1 Baseline characteristics of validation population

| Characteristics | Levels | Non-gastric cancer (N=210) | Gastric cancer (N=81) | p |
| --- | --- | --- | --- | --- |
| Sex (%) | Male | 172 (81.9) | 61 (75.3) | 0.272 |
|  | Female | 38 (18.1) | 20 (24.7) |  |
| Age (years) (%) | <60 | 137 (65.2) | 28 (34.6) | <0.001 |
|  | ≥60 | 73 (34.8) | 53 (65.4) |  |
| BMI (%) | <18.5 | 0 (0.0) | 3 (3.7) | 0.019 |
|  | 18.5-23.9 | 88 (41.9) | 32 (39.5) |  |
|  | ≥23.9 | 122 (58.1) | 46 (56.8) |  |
| Marriage (%) | Unmarried | 8 (3.8) | 1 (1.2) | 0.448 |
|  | Married | 202 (96.2) | 80 (98.8) |  |
| Education (%) | Primary and below | 167 (79.5) | 44 (54.3) | <0.001 |
|  | Secondary school | 30 (14.3) | 26 (32.1) |  |
|  | College and above | 13 (6.2) | 11 (13.6) |  |
| Occupational exposure (%) | Absent | 129 (61.4) | 62 (76.5) | 0.022 |
|  | Present | 81 (38.6) | 19 (23.5) |  |
| Smoking status (%) | Never smoker | 112 (53.3) | 45 (55.6) | 0.002 |
|  | Smoker | 95 (45.2) | 28 (34.6) |  |
|  | Ex-smoker | 3 (1.4) | 8 (9.9) |  |
| Drinking status (%) | Absent | 143 (68.1) | 57 (70.4) | 0.815 |
|  | Present | 67 (31.9) | 24 (29.6) |  |
| Vegetables (%) | Seldom | 35 (16.7) | 20 (24.7) | 0.162 |
|  | Frequently | 175 (83.3) | 61 (75.3) |  |
| Fruits (%) | Seldom | 66 (31.4) | 40 (49.4) | 0.007 |
|  | Frequently | 144 (68.6) | 41 (50.6) |  |
| Milk, meat and egg production (%) | Seldom | 175 (83.3) | 61 (75.3) | 0.162 |
|  | Frequently | 35 (16.7) | 20 (24.7) |  |
| Pickled food (%) | Seldom | 158 (75.2) | 60 (74.1) | 0.957 |
|  | Frequently | 52 (24.8) | 21 (25.9) |  |
| Fried food (%) | Seldom | 178 (84.8) | 62 (76.5) | 0.139 |
|  | Frequently | 32 (15.2) | 19 (23.5) |  |
| Hot diet (%) | Seldom | 157 (74.8) | 49 (60.5) | 0.024 |
|  | Frequently | 53 (25.2) | 32 (39.5) |  |
| History of UDTDs (%) | Absent | 174 (82.9) | 46 (56.8) | <0.001 |
|  | Present | 36 (17.1) | 35 (43.2) |  |
| Diabetes (%) | Absent | 203 (96.7) | 68 (84.0) | <0.001 |
|  | Present | 7 (3.3) | 13 (16.0) |  |
| Hypertension (%) | Absent | 194 (92.4) | 46 (56.8) | <0.001 |
|  | Present | 16 (7.6) | 35 (43.2) |  |
| Hyperlipemia (%) | Absent | 194 (92.4) | 56 (69.1) | <0.001 |
|  | Present | 16 (7.6) | 25 (30.9) |  |
| Family history of cancer (%) | Absent | 161 (76.7) | 39 (48.1) | <0.001 |
|  | Present | 49 (23.3) | 42 (51.9) |  |
| Family history of gastric cancer (%) | Absent | 202 (96.2) | 61 (75.3) | <0.001 |
|  | Present | 8 (3.8) | 20 (24.7) |  |
| H.Pylori (%) | Negative | 199 (94.8) | 66 (81.5) | 0.001 |
|  | Positive | 11 (5.2) | 15 (18.5) |  |
| KRT7 (%) | Negative | 114 (54.3) | 35 (43.2) | 0.118 |
|  | Positive | 96 (45.7) | 46 (56.8) |  |
| DKK3 (%) | Negative | 69 (32.9) | 50 (61.7) | <0.001 |
|  | Positive | 141 (67.1) | 31 (38.3) |  |
| SERPINB5 (%) | Negative | 127 (60.5) | 15 (18.5) | <0.001 |
|  | Positive | 83 (39.5) | 66 (81.5) |  |

BMI: body mass index; History of UDTSs: History of upper digestive tract diseases; H.Pylori: Helicobacter pylori; KET 7: Keratin 7; DKK 3: Dickkopf-associated protein 3; SERPINB5: Mammary fibrostatin
